# Ethical and regulatory issues of pragmatic cluster randomized trials in contemporary health systems

**DOI:** 10.1177/1740774515571140

**Published:** 2015-03-01

**Authors:** Monique L Anderson, Robert M Califf, Jeremy Sugarman

**Affiliations:** 1 Division of Cardiology, Department of Medicine, Duke University School of Medicine, Durham, North Carolina; 2 Duke Translational Medicine Institute, Duke University, Durham, North Carolina; 3 Berman Institute of Bioethics, Johns Hopkins University, Baltimore, Maryland; 4 Department of Medicine, Johns Hopkins School of Medicine, Baltimore, Maryland

## Abstract

Cluster randomized trials (CRTs) randomly assign groups of individuals to examine research questions or test interventions and measure their effects on individuals. Recent emphasis on quality improvement, comparative effectiveness, and learning health systems has prompted expanded use of pragmatic CRTs in routine healthcare settings, which in turn poses practical and ethical challenges that current oversight frameworks may not adequately address. The 2012 Ottawa Statement provides a basis for considering many issues related to pragmatic CRTs but challenges remain, including some arising from the current U.S. research and healthcare regulations. In order to examine the ethical, regulatory, and practical questions facing pragmatic CRTs in healthcare settings, the National Institutes of Health (NIH) Health Care Systems Research Collaboratory convened a workshop in Bethesda, Maryland in July of 2013. Attendees included experts in clinical trials, patient advocacy, research ethics, and research regulations from academia, industry, the NIH, and other federal agencies. Workshop participants identified substantial barriers to implementing these types of CRTs, including issues related to research design, gatekeepers and governance in health systems, consent, institutional review boards, data monitoring, privacy, and special populations. We describe these barriers and suggest means for understanding and overcoming them to facilitate pragmatic CRTs in healthcare settings.

## Introduction and background

Cluster-randomized trials (CRTs), also known as group-randomized trials, randomly assign groups of individuals within a population of interest, such as people in a particular research site, clinic, hospital, or city block,^1–3^ and measure the effects of an intervention at the individual level.^4^ In contrast to individual randomization, cluster randomization permits the evaluation of a cluster-level intervention, may be logistically simpler and less expensive than a conventional randomized trial, and can help reduce the effect of treatment contamination.^2,5^ Once largely confined to public health and education research, CRTs are now employed across a widening array of healthcare settings.^6,7^ Given the current emphasis on quality improvement, comparative effectiveness research,^8^ and learning health systems,^9^ the deployment of *pragmatic* CRTs in healthcare settings will likely continue to expand. Pragmatic clinical trials have been defined as

“*…trials for which the hypothesis and study design are formulated based on information needed to make a decision….The most distinctive features of PCTs [pragmatic clinical trials] are that they select clinically relevant interventions to compare, include a diverse population of study participants, recruit participants from a variety of practice settings, and collect data on a broad range of health outcomes*.”^10^

Indeed, pragmatic CRTs may be ideal for addressing the types of issues evaluated in health systems, especially when various accepted practices are evaluated.^11^ However, the fundamental difference in the unit of randomization compared with individually randomized trials, together with the fact that data collection can occur at multiple levels (e.g., patient, clinician, and facility) make the ethical and regulatory review of pragmatic CRTs in clinical settings especially challenging,^11,12^ partly because existing research and clinical oversight frameworks were largely developed without such trials in mind.^13–17^

The 2012 Ottawa Statement on the Ethical Design and Conduct of Cluster Randomized Trials^17^ identifies important issues related to CRTs in general and offers recommendations at the intersection of ethics and CRTs (Table 1). Although the Ottawa Statement provides a substantial foundation for understanding many of the ethical issues associated with CRTs, additional ethical, regulatory, and practical challenges remain.^18^ Some of these issues relate specifically to the use of pragmatic CRTs in healthcare settings within the current U.S. regulatory system governing research and medical practice.

## The collaboratory workshop on cluster-randomized trials

The National Institutes of Health Care Systems Research Collaboratory (NIH Collaboratory; https://www.nihcollaboratory.org/Pages/default.aspx) seeks to facilitate pragmatic clinical trials, including CRTs when appropriate, with the overarching goal of substantially increasing the pool of high-quality, generalizable evidence to guide decisions about health and healthcare while concomitantly reducing research costs. Of seven trials initially funded for a year-long planning phase in the NIH Collaboratory, five were CRTs (Table 2).

In order to examine the ethical, regulatory, and practical issues posed by pragmatic CRTs in healthcare settings, the Collaboratory convened a day-long workshop in Bethesda, Maryland in July of 2013. Participants were identified from those working in the Collaboratory and beyond based upon a review of literature related to CRTs, research ethics, and research oversight (Appendix), and were selected to ensure balanced expert representation from academia (including biostatisticians and clinical trialists with expertise in cluster-randomized trials and ethicists), research funding bodies, regulatory agencies, and the medical products industry. The workshop was a moderated roundtable forum in which participants were solicited to provide brief prepared presentations, followed by discussion among all participants. Although the meeting itself did not incorporate a formal process for polling or otherwise establishing formal consensus, a draft version of this paper was furnished to all workshop participants for their review and comments.

The goals of the workshop were to 1) foster dialogue that could help build a consensus in the future around best practices for pragmatic CRTs and 2) identify areas needing further research and policy deliberations regarding pragmatic CRTs both within and beyond the United States. Workshop participants understood that although pragmatic CRTs have the potential to powerfully address a range of important health-related issues, there are substantial barriers to implementing them in clinical settings. Meeting discussions identified multiple challenges to the successful implementation of pragmatic CRTs, which fall into the following categories: 1) research design; 2) gatekeepers and governance in health systems; 3) consent; 4) institutional review boards (IRBs); 5) data monitoring; 6) privacy; and 7) special populations. Although some of these issues necessarily overlap with the ethics and regulatory challenges in general that have been described elsewhere,^19^ here we discuss each of these as they relate to pragmatic CRTs in particular, and where possible, address potential means for better understanding and overcoming these challenges.

### Research design

CRTs pose fundamental questions about who should be considered a research subject,^20,21^ which in turn raises concerns about risks, benefits, and consent. For example, if groups of clinicians are randomly assigned to an educational intervention and their patients are the recipients of advice and decisions by clinicians exposed to the intervention, who is rightly considered the “subject” of research? While clinicians’ behavior may be monitored, a meaningful pragmatic CRT will measure clinical outcomes that are directly related to patients’ well-being. Thus, it can be difficult to determine who in fact is a research subject: Patients? Clinicians? Both? For example, in the Collaboratory’s Active Bathing to Eliminate Infection (ABATE) Trial, hospitals are randomly assigned in a factorial design to daily bathing and/or mupirocin versus usual care for patients at high risk for multidrug-resistant *Staphylococcus aureus*.^22^ In this trial, nurses administer the interventions, but should they also be considered to be research participants? What about patients’ visitors, who may also be at risk of infection?

Making such a determination is important as an ethical matter for ensuring appropriate protections tailored to the risks and burdens imposed by the research. Similarly, making such determinations can involve important regulatory considerations. Under the current U.S. regulatory framework, a “human subject” is defined as “a living individual about whom an investigator (whether professional or student) conducting research obtains 1) Data through intervention or interaction with the individual, or 2) Identifiable private information.”^23^ Although this definition would include many of those affected by a CRT, it does not necessarily capture them all, especially if the interventions are conducted on distinct populations from whom data are collected. McRae and colleagues propose that a “research subject is an individual whose interests may be compromised as a result of interventions in a research study.”^21^ They further specify four criteria for identifying research subjects:

“*1. An individual who is directly intervened upon by an investigator; 2. An individual who is deliberately intervened upon via manipulation of the individual’s environment by an investigator; 3. An individual with whom an investigator interacts for the purpose of collecting data; or, 4. An individual about whom an investigator obtains identifiable private information for the purpose of collecting data*.”^21^

### Gatekeepers and governance in health systems

A gatekeeper is “a person who controls access.”^24^ In the context of pragmatic CRTs in health systems this may include administrators or clinicians who can permit or foreclose the possibility of conducting such a trial within the system. For example, the Collaboratory-sponsored Time to Reduce Mortality in End-stage Renal Disease (TIME) Trial^25^ is randomly assigning dialysis units to standard dialysis regimes versus 4.5 hours of dialysis as a general strategy, with individual patients allowed to opt out of the study. Fundamentally, this trial was feasible only because two companies that own dialysis units decided to permit it to take place within their respective units. The executives of these businesses effectively functioned as gatekeepers.

In many types of CRTs, gatekeepers may have the authority to allow researchers to directly approach participants or give permission to enroll the cluster in a study.^26^ This does not imply that gatekeepers have authority to provide consent for individual participants when such consent is necessary; rather, gatekeepers are generally charged with advancing the interests of a cluster and preserving its trust.^11^ In health systems, the role and breadth of authority of gatekeepers who are in a position to allow or prohibit particular CRTs are complex and poorly defined. For example, in such settings, gatekeepers may include locally practicing physicians, a managing partner of a primary care practice, hospital administrative leadership, and/or health systems-level leadership.^27^ Because a single gatekeeper may have significant conflicts of interest and/or obligations in protecting cluster interests, multiple gatekeepers (of whom a health system would be expected to have at least several) may be needed to provide consent to *cluster* participation. However, as a practical matter, it may be difficult or impossible to truly identify all explicit and tacit gatekeepers, particularly in the setting of large-scale pragmatic CRTs. This suggests the need for more carefully articulated governance models for CRTs in health systems.^15,26^

Gallo and colleagues provide a framework for understanding the nature of gatekeepers in CRTs. Their framework sees a limited role for gatekeepers in terms of obtaining consent from them for cluster randomization. For instance, they suggest that if consent from participants will be obtained after randomization and before intervention, then gatekeeper consent is not required.^26^ However, gatekeepers can protect the interests of the cluster in other ways, especially when organizational interests are affected by CRTs.^26^

Currently, generally accepted governance models specific to pragmatic CRTs in healthcare settings do not exist. However, elements of such a model would likely include representation by members of the cluster and mechanisms of decision-making that are transparent as well as sensitive to the culture(s) and values of the clusters. Organizational oversight in health systems might include a research advisory committee (to serve as a gatekeeper protecting cluster interests), a scientific review committee, an IRB liaison to facilitate determination about whether IRB oversight is required for a particular CRT, and a clinical oversight committee (whose members review study data and incorporate findings into clinical care).^28^

### Consent: ethical considerations

As mentioned above, permission to approach a cluster for inclusion in a CRT, perhaps granted by gatekeepers or through a governance process, is separable from express consent to participate that is obtained subsequently from individuals, should it be required. In some CRTs, individual consent remains feasible and appropriate. For example, a pragmatic CRT might assess the provision of aggressive physical therapy for surgical rehabilitation compared to usual care. Patients assigned to the active arm might need to devote considerable time to the program, which may obviate their ability to engage in other activities. In such studies, individual consent arguably should be obtained.

However, pragmatic CRTs can involve randomly assigning clinic systems or hospitals, where obtaining express individual consent is not meaningful, because the entire cluster will be affected by the randomization and there are no particular choices for individuals to make. Such pragmatic CRTs bear a closer relationship to CRTs in community settings. For example, in the ABATE pragmatic CRT, which is testing different bathing practices to assess the best means of decreasing nosocomial infections,^22^ there may not be any alternatives (because the health system has applied it in standard fashion to all patients) nor any burdens or risks associated with data that are routinely collected (because data are routinely collected as a part of patient care) and analyzed to assess this particular question.

Further, in other pragmatic CRTs it may not be feasible to obtain consent for the cluster-level intervention, yet it may still be feasible to notify those affected about what is being evaluated and about available alternatives, as well as to obtain permission for uses of data. Thus, the nature of the interventions being tested plays a critical role in determining whether consent should be obtained and when other means of disclosure and authorization might be employed. Several of the NIH Collaboratory’s trials are not obtaining express written consent; in these trials, the responsible IRBs have determined that the relevant regulatory requirements for such an approach have been satisfied. A description of these issues in each of the Collaboratory’s trials can be found at the Collaboratory website.^29^

A further issue concerns the need for obtaining consent in different arms of a CRT. For example, it could be argued that consent is unnecessary from members of the control group, who receive usual care and for whom data are collected within the context of routine health care (for which mechanisms are in place to protect personal health information).^30^ However, assuming that the experimental group receives significantly altered care with more than minimal risk, those randomized to the intervention arm clearly must be asked to provide consent. Nevertheless, such an approach may lead to a consent bias due to an asymmetry of agreement to participate, possibly compromising the trial’s integrity.

Workshop participants generally felt the Ottawa Statement was too restrictive in its determination of when it is appropriate to waive or alter informed consent. In particular, the Statement recommends that researchers obtain consent except when “(1) the research is not feasible without a waiver or alteration of consent, and (2) the study interventions and data collection procedures pose no more than minimal risk.”^17^ However, some comparative-effectiveness research (which often compares standard-of-care interventions) and quality improvement projects that employ cluster randomization may pose situations in which prospective informed consent for particular activities may not seem appropriate. Moreover, requiring consent in these settings may preclude a large number of important and well-designed trials that involve low incremental risk, resulting in interventions being adopted or discontinued without meaningful information regarding their value.

While participants’ rights and interests must be respected, upholding such obligations may be possible using alternatives of notification and authorization to conventional informed consent procedures, such as informing participants of the study and giving them the ability to opt out without penalty. Although federal research regulations permit such approaches in limited circumstances as described below, they are precluded for research that involves greater than minimal risk (there are special provisions for some research in emergency settings and in certain types of demonstration projects).

### Consent: regulatory considerations

Under U.S. Department of Health and Human Services regulations for research with human subjects—i.e., the “Common Rule,”^31^ so called because of its widespread adoption by other federal agencies (but, importantly, not the U.S. Food and Drug Administration [FDA]), as discussed below)—criteria for waiving or modifying consent in non-emergency settings require that:

“*1) the research involves no more than minimal risks to the subjects; 2) the waiver or alteration will not adversely affect the rights or welfare of the subjects; 3) the research could not be practicably carried out without the waiver or alteration; and 4) whenever appropriate, the subjects will be provided with additional pertinent information after participation*.”^31^

Of note, these federal regulations define “minimal risk” as “…the probability and magnitude of harm or discomfort anticipated in the research are not greater in and of themselves than those ordinarily encountered in daily life or during the performance of routine physical or psychological examinations or tests.”^32^

However, among IRBs there can be disagreements about determining whether a particular CRT entails no more than minimal risk—a key criterion for permitting a waiver of consent that may be important to ensuring trial integrity.^33–35^ For pragmatic CRTs conducted in health systems, debates about minimal risk for a particular trial (perhaps one involving elements of usual care) can be confounded by how minimal risk is defined and whether the research poses incremental risk. Clarification of how these federal regulations should be properly interpreted for pragmatic clinical trials involving standard-of-care interventions are clearly needed to facilitate the appropriate conduct of these types of these trials. Draft guidance on this issue has been released recently by the Office for Human Research Protections^36^; however, it has proven controversial.^37^

Although a comprehensive discussion of this issue (itself not unique to CRTs) is beyond the scope of this article, some confusion can be resolved by attending to the criteria that IRBs are supposed to use in making their determinations.^15^ Specifically, according to the Common Rule: “In evaluating risks and benefits, the IRB should consider only those risks and benefits that may result from the research (as distinguished from risks and benefits of therapies subjects would receive even if not participating in the research).”^38^

There may also be significant regulatory barriers to the conduct of pragmatic CRTs in health systems when they involve drugs, devices, or biologics that either fall under the purview of FDA regulations or include FDA-approved products. Unlike the Common Rule governing non-FDA-regulated research described above, which includes mechanisms for waiving or modifying consent, FDA regulations make limited provisions for modifying the informed consent process except for some research in emergency research settings^39^ and allow *documentation* of consent to be waived only when an IRB determines “the research presents no more than minimal risk and involves no procedures for which written consent is normally required outside the research context.”^40^ Further, some workshop participants indicated that the FDA generally views even studies involving approved drugs as “clinical investigations” that should not be considered minimal risk. This informal policy, which can influence the determination of risk by IRBs, may inadvertently hamper CRTs that include FDA-regulated products in healthcare settings. Clarification is needed regarding whether trials comparing medical products whose labeled use is considered standard care fall under the purview of the FDA, and if so, whether these trials would be considered minimal risk. In the cases of the ABATE^22^ and the TIME trials,^25^ the judgment of relevant parties was that the trials did not fall under FDA purview although medical products were involved.

### Institutional review boards

Institutional review boards play a critical role in determining whether pragmatic CRTs are acceptable in specific health systems. Currently, IRBs may make variable determinations regarding pragmatic CRTs, including whether consent may be required for a particular trial. As described below, they must also consider the relevant regulations regarding “vulnerable subjects” such as children, prisoners, and pregnant women. In order to properly navigate the regulatory complexities associated with pragmatic CRTs, IRBs would benefit from guidance from the Office for Human Research Protections and FDA regarding the interpretation of relevant regulations. In addition, most IRBs would likely benefit from educational programs regarding the practical, ethical, and regulatory issues relevant to CRTs that are described in this article.

Because pragmatic CRTs typically involve multiple research sites, appropriate alignment of IRB functions is critical for harmonization and efficiency across site institutions. Although major decisions regarding the design and conduct of a CRT might occur at a primary or central IRB, local input is vital for context and oversight of local implementation of a trial at each research site. IRBshare (irbshare.org), which offers shared review documents and processes used in traditional multisite randomized controlled trials, may provide a mechanism by which IRBs can participate in protocol review and updates on the protocol as a joint activity, while still maintaining local oversight of pragmatic CRTs. To be most effective, this centralized process should include a panel with expertise in pragmatic CRTs.

### Data monitoring

The nature of CRTs in health systems can raise practical and conceptual challenges with respect to data monitoring, which aims to ensure data integrity and the well-being of participants. At a practical level, consider data monitoring for traditional randomized trials compared with pragmatic CRTs. In the former, an orderly collection of data is typically planned for each individual study participant. Summary information is then forwarded to a data coordinating center and periodically reviewed by a data monitoring committee. In contrast, pragmatic CRTs are increasingly harvesting data at intervals from a central source or use query systems in which individual data are not aggregated except as needed for specific purposes, thus necessitating different approaches to data monitoring. However, because many pragmatic CRTs test the effectiveness of medical products or strategies in a manner that could result in major differences in morbid and mortal outcomes in the randomized populations, it would be prudent to have a mechanism such as data monitoring committees to monitor these trials in timely fashion for evidence of clear benefit or harm. The initial round of Collaboratory trials have initiated various data and safety monitoring procedures, ranging from full traditional data monitoring committees in trials evaluating morbid and mortal outcomes to using a smaller number of reviewers for lowest-risk trials.^29^

CRTs also raise complex statistical issues, prompting questions about when it might be appropriate to stop a trial due to apparent efficacy, futility, or safety concerns.^13^ Accordingly, when developing monitoring plans for CRTs, it is essential that experts with relevant statistical knowledge are engaged not only in study development and analyses but also in monitoring.

### Privacy

The Health Insurance Portability and Accountability Act (HIPAA) Privacy Rule,^41^ which establishes privacy protections for medical records and other protected health information within covered entities, deserves special consideration in the context of pragmatic CRTs conducted in US healthcare settings. There is currently little literature that examines the application of HIPAA to these trials. Some CRTs may be eligible for an alteration or waiver of HIPAA authorization if the activity 1) poses no more than minimal risk to individual privacy; 2) cannot be conducted without a waiver; and 3) cannot be conducted without access to protected health information.^42^ If these conditions are not met, full authorization must be obtained unless de-identified or limited data sets can be used, which may not be ideal for many pragmatic CRTs.

For research that meets criteria for a waiver or alteration of authorization, “opt-out” procedures may offer a relatively simple process that likely would not compromise the inclusion of a representative population.^43^ Some registries have used business associate agreements that permit data to be gathered and shared for quality improvement purposes. Under such agreements, aggregated data can be de-identified and used for research.^44^ Pragmatic CRTs within healthcare settings could explore the feasibility of such an approach in situations where it would be acceptable to analyze de-identified data from clusters. However, this may be especially problematic for research under the purview of the FDA, for which individual-level data will likely be necessary.

### Special populations

Enrolling certain populations in pragmatic CRTs in healthcare settings may be accompanied by additional regulatory and ethical considerations to afford special protections to “vulnerable subjects”. In the U.S., federal regulations in this regard include provisions for research involving children, pregnant women, and prisoners. The relevant regulations differ in important ways, but each necessitates that IRBs make special determinations, and there can be particular provisions for consent. Although a full exploration of these issues is beyond the scope of this paper, making such determinations and provisions across broad clusters may be considerably complex, especially with pragmatic CRTs that span health systems.

The Collaboratory’s Suicide Prevention Trial^45^ is an interesting example in which vulnerable subjects—depressed and potentially suicidal patients—are approached for trial participation. In this case the investigators worked with their IRB and the National Institute of Mental Health to conduct an iterative series of pilot studies to find a suitable method for consent and participation.

As a related matter, within healthcare systems, attention should be given to ensuring that healthcare professionals are not forced to allow their clinics, practices, or wards to be included in a particular PCT. With this in mind, as mentioned in the Ottawa Statement, “consent negotiations should be conducted without the presence of cluster or organization leaders, and cluster or organizational leaders should not be informed of the identities of those who agree to or decline study participation.”^17^

## Strengths and limitations

This article distills the expert knowledge, experience and opinions of a wide array of workshop participants, incorporating diverse perspectives from across the spectrum of ethics, research, clinical care, funding bodies, and regulators. As such, it outlines a set of important topics and issues that warrant attention in the context of pragmatic CRTs. While widespread agreement exists in some areas as described above, others require additional scholarship (both conceptual and empirical) and deliberation. The manuscript was disseminated to all 37 conference participants, of whom 24 provided comments or suggested editorial changes. One participant declined to review the manuscript because of a potential conflict of interest as an Ottawa Statement co-author.

## Next steps

As depicted in Table 3, broad implementation of pragmatic CRTs within contemporary health systems will require collaboration among many sectors. Although there may be no absolutely “correct” solution to these issues, there is a need for guidance that enables health systems and researchers to increase the body of reliable evidence to drive safe, effective, and efficient clinical care while also protecting the rights and choices of individuals and populations. Ongoing empirical ethics studies will give us a deeper understanding of the views of patients and families and of administrators and providers. In the coming years, the Collaboratory plans to address each of these issues in more detail, convening additional expert groups and public input to provide detailed insight needed to achieve a workable consensus.

## Figures and Tables

**Table 1:** Summary of recommendations from the Ottawa Statement*

Ethical issue	Number	Recommendation
Justifying CRT design		
	1	Researchers should provide a clear rationale for the use of the cluster randomized design and adopt statistical methods appropriate for this design.
REC review		
	2	Researchers must submit a CRT involving human research participants for approval by a REC before commencing.
Identifying research participants		
	3	Researchers should clearly identify the research participants in CRTs. A research participant can be identified as an individual whose interests may be affected as a result of study interventions or data collection procedures, that is, an individual (1) who is the intended recipient of an experimental (or control) intervention; or (2) who is the direct target of an experimental (or control) manipulation of his/her environment; or (3) with whom an investigator interacts for the purpose of collecting data about that individual; or (4) about whom an investigator obtains identifiable private information for the purpose of collecting data about that individual. Unless one or more of these criteria is met, an individual is not a research participant.
Obtaining informed consent		
	4	Researchers must obtain informed consent from human research participants in a CRT, unless a waiver of consent is granted by a REC under specific circumstances.
	5	When participants' informed consent is required, but recruitment of participants is not possible before randomization of clusters, researchers must seek participants' consent for trial enrollment as soon as possible after cluster randomization—that is, as soon as the potential participant has been identified, but before the participant has undergone any study interventions or data collection procedures.
	6	A REC may approve a waiver or alteration of consent requirements when (1) the research is not feasible without a waiver or alteration of consent, and (2) the study interventions and data collection procedures pose no more than minimal risk.
	7	Researchers must obtain informed consent from professionals or other service providers who are research participants unless conditions for a waiver or alteration of consent are met.
Gatekeepers		
	8	Gatekeepers should not provide proxy consent on behalf of individuals in their cluster.
	9	When a CRT may substantially affect cluster or organizational interests, and a gatekeeper possesses the legitimate authority to make decisions on the cluster or organization's behalf, the researcher should obtain the gatekeeper's permission to enroll the cluster or organization in the trial. Such permission does not replace the need for the informed consent of research participants, when it is required.
	10	When CRT interventions may substantially affect cluster interests, researchers should seek to protect cluster interests through cluster consultation to inform study design, conduct, and reporting. Where relevant, gatekeepers can often facilitate such a consultation.
Assessing benefits & harms		
	11	The researcher must ensure that the study intervention is adequately justified. The benefits and harms of the study intervention must be consistent with competent practice in the field of study relevant to the CRT.
	12	Researchers must adequately justify the choice of the control condition. When the control arm is usual practice or no treatment, individuals in the control arm must not be deprived of effective care or programs to which they would have access, were there no trial.
	13	Researchers must ensure that data collection procedures are adequately justified. The risks associated with data collection procedures must (1) be minimized consistent with sound design and (2) stand in reasonable relation to the knowledge to be gained.
Protecting vulnerable participants		
	14	Clusters may contain vulnerable participants. In these circumstances, researchers and RECs must consider whether additional protections are needed.
	15	When individual informed consent is required and there are individuals who may be less able to choose participation freely because of their position in a cluster or organizational hierarchy, RECs should pay special attention to recruitment, privacy, and consent procedures for those participants.

* Reproduced from Table 1 (“Summary of Recommendations”) in Weijer C, Grimshaw JM, Eccles MP, McRae AD, White A, et al. *PLoS Med* 2012; 9: e1001346.

**Table 2:** Funded 2012 NIH Health Care Systems Research Collaboratory demonstration projects incorporating cluster designs (UH2)

Demonstration project	Lead institution/organization	Participating institutions	Randomization level	Planned consent
A Pragmatic Trial of Lumbar Image Reporting with Epidemiology (LIRE) (NCT02015455)	University of Washington	KP Northern California, Group Health Cooperative, Mayo Clinic, Henry Ford Health System	Clinics	Alteration/Waiver
Collaborative Care for Chronic Pain in Primary Care (NCT01888146)	Kaiser Permanente Health Systems	KP Hawaii, KP Georgia, KP Northwest	Clinics	Alteration/Waiver
Active Bathing to Eliminate Infection Project (ABATE) (NCT02063867)	University of California,	Hospital Corporation of America, Harvard Pilgrim Health Care	Hospitals	Alteration/Waiver
Strategies and Opportunities to Stop Colon Cancer in Priority Populations (Stop CRC) (NCT01742065)	Kaiser Foundation Research Institute	Oregon Community Health Information Network (OCHIN) Federally Qualified Health Center Clinics	Federally Qualified Health Centers	Alteration/Waiver
Time to Reduce Mortality in End-Stage Renal Disease (TiME) Trial (NCT02019225)	University of Pennsylvania	Fresenius Medical Care North America; DaVita	Dialysis Centers	Alteration/Waiver

**Table 3:** Key issues in deploying pragmatic cluster randomized trials in contemporary health systems

	Issue type				
Domains	Ethical	Regulatory	Practical	Responsible parties	Next steps
Trial design					Guidance
Subject identification	X	X	X	FDA, NIH, ORHP	
Indirect participant/bystander	X		X	Ethicists, trialists	Scholarship
Gatekeepers and governance					
Gatekeepers	X		X	Healthcare system leaders, research and/or clinical oversight committees, ethicists	Research to include stakeholder perspectives, scholarship, develop and evaluate governance models
Governance	X		X		
Consent	X	X		Ethicists, FDA, NIH, OHRP	Guidance and policy development, research with stakeholders, scholarship, and evaluation
*Ethical aspects*					
Feasibility	X	X	X		
Differential risk/benefit balance in arms	X	X	X		
*Regulatory considerations*					
When can consent be waived?	X	X			
Determination of minimal risk	X	X			
FDA regulation		X			
Institutional review boards				HHS, OHRP, IRBs	Guidance and policy development, curriculum development and implementation
Education	X	X			
Local, shared, and central review	X	X	X		
Data monitoring				Ethicists, statisticians, DMC experts	Scholarship, guidance development
Use in CRTs	X		X		
Stopping rules	X		X		
Data sources for interim analysis	X		X		
Privacy		X		Office of Civil Rights, ethicists, CRT experts	Policy development
Application to CRTs		X			
Alteration or waiver Use		X			
Gatekeepers	X		X		
Special populations				Ethicists, FDA, NIH, OHR, CRT Researchers	Guidance and policy development; think tanks, public hearings
Vulnerable populations	X	X	X		

CRT: cluster-randomized trial; HHS: U.S. Department of Health and Human Services; DMC: data monitoring committee; FDA: U.S. Food and Drug Administration; IRB: institutional review board; NIH: National Institutes of Health; OHRP: Office for Human Research Protections

## Appendix

**Table:** Participants in the Health Care Systems Research Collaboratory’s Workshop on Cluster Randomized Trials: Ethical, Regulatory, and Practical Issues

Name	Title and Affiliation
Monique L. Anderson, MD*	Medical Instructor, Division of Cardiology, Department of Medicine, Duke University School of Medicine
Patrick Archdeacon, MD	Medical Officer, Office of Medical Policy, Center for Drug Evaluation and Research, Food and Drug Administration
Mark Barnes, JD, LLM	Co-Director, Multi-Regional Clinical Trials Center, Harvard University
Laura M. Beskow, MPH, PhD*	Associate Professor, Duke Clinical Research Institute and Faculty Associate, Trent Center for Bioethics, Humanities & History of Medicine
Barbara E. Bierer, MD*	Senior Vice President for Research, Brigham Research Institute, and Director, Center for Faculty Development and Diversity, Brigham and Women's Hospital; Harvard Medical School
Robert Califf, MD*	Director, Duke Translational Medicine Institute and Vice Chancellor for Clinical & Translational Research, Duke University School of Medicine
Rowena Dolor, MD, MHS*	Director, Duke Primary Care Research Consortium, Duke Translational Medicine Institute
Allan Donner, PhD±	Professor, Department of Epidemiology and Biostatistics, Western University
Susan Ellenberg, PhD*	Senior Scholar, Center for Clinical Epidemiology & Biostatistics – Department of Biostatistics and Epidemiology, and Professor of Biostatistics, University of Pennsylvania Perelman School of Medicine
M. Khair ElZarrad, PhD, MPH*	Science Policy Analyst, National Institutes of Health
Rachael Fleurence, PhD, MSc	Science Program Director, CER Methods and Infrastructure, Patient-Centered Outcomes Research Institute
David Forster, JD, MA*	Chief Compliance Officer, WIRB-Copernicus Group; University of Washington
Sara Goldkind, MD, MA*	Senior Bioethicist, Office of Compliance, Food and Drug Administration
Steve Goodman, MD, PhD, MHS	Chief of the Division of Epidemiology, Department of Health Research and Policy; Co-Director, Meta-Research Innovation Center at Stanford, Stanford University
R. Peter Iafrate, PharmD*	Chairman, Institutional Review Board, University of Florida Health Science Center
Nancy Kass, ScD	Professor of Bioethics and Public Health, Department of Health Policy and Management, Johns Hopkins Bloomberg School of Public Health; Deputy Director for Public Health, Berman Institute of Bioethics
John M. Kessler, PharmD*	Adjunct Associate Professor, UNC Eshelman School of Pharmacy; Chief Clinical Officer, SecondStory Health, LLC.
Michael Lauer, MD	Director, Division of Cardiovascular Sciences, National Heart, Lung, and Blood Institute, National Institutes of Health
Joanne Less, PhD*	Director, Office of Good Clinical Practice, Food and Drug Administration
Leanne Madre, JD, MHA*	Director of Strategy, Clinical Trials Transformation Initiative, Duke Translational Medicine Institute
Diane Maloney, JD	Associate Director for Policy, Center for Biologics Evaluation and Research, Food and Drug Administration
Rachael Moloney, MHS*	Research Manager, Center For Medical Technology Policy
Ross McKinney, MD	Director, Trent Center for Bioethics, Humanities & History of Medicine, Duke University; Professor of Pediatric Infectious Diseases, Duke University School of Medicine
Jerry Menikoff, MD, JD*	Director, Office for Human Research Protections, U.S. Department of Health and Human Services
David M. Murray, PhD*	Director, Office of Disease Prevention, Office of the Director, National Institutes of Health
Jane Perlmutter, PhD, MBA	Founder, Gemini Group
Richard Platt, MD, MS*	Professor and Chair, Department of Population Medicine, Harvard Pilgrim Health Care Institute
Ivor Pritchard, PhD*	Senior Advisor to the Director, Office for Human Research Protections, U.S. Department of Health and Human Services
Jim Sabin, MD	Director, Ethics Program, Harvard Pilgrim Health Care Institute; Clinical Professor, Population Medicine and Psychiatry, Harvard Medical School
Rachel Sherman, MD. MPH	Former Director, Center for Drug Evaluation and Research, Food and Drug Administration; Principal, Drug and Biological Drug Products, Greenleaf Health, LLC
Jeremy Sugarman, MD, MPH, MA*	Deputy Director for Medicine, Johns Hopkins Berman Institute of Bioethics; Professor of Bioethics and Medicine, Johns Hopkins University
Robert Temple, MD*	Deputy Director for Clinical Science, Center for Drug Evaluation and Research, Food and Drug Administration
Pamela Tenaerts, MD, MBA*	Director, Clinical Trials Transformation Initiative, Duke Translational Medicine Institute
Wendy Weber, ND, PhD, MPH*	Branch Chief, Clinical Research in Complementary and Integrative Health Branch, National Center for Complementary and Alternative Medicine, National Institutes of Health
Kevin Weinfurt, PhD	Professor in Psychiatry and Behavioral Sciences, Duke University
David Wendler, PhD*	Head, Unit on Vulnerable Populations, Department of Bioethics, Clinical Center, National Institutes of Health
Ashley Wivel, MD, MSc*	Senior Director, Clinical Effectiveness and Safety, GlaxoSmithKline
David Zhang, PhD	Global Lead, External Collaborations and Strategic Partnership,
	Roche

* provided feedback on the manuscript

± conflict of interest, Ottawa co-author
